# Druggable gene alterations in Japanese patients with rare malignancy

**DOI:** 10.1016/j.neo.2022.100834

**Published:** 2022-09-08

**Authors:** Akihiro Ohmoto, Naomi Hayashi, Ippei Fukada, Masumi Yamazaki, Mayu Yunokawa, Akiyoshi Kasuga, Eiji Shinozaki, Arisa Ueki, Akiko Tonooka, Kengo Takeuchi, Seiichi Mori, Kazuma Kiyotani, Shunji Takahashi

**Affiliations:** a Division of Medical Oncology, Cancer Institute Hospital of Japanese Foundation for Cancer Research, Tokyo, Japan; b Division of Genomic Medicine, Cancer Institute Hospital of Japanese Foundation for Cancer Research, Tokyo, Japan; c Division of Breast Medical Oncology, Cancer Institute Hospital of Japanese Foundation for Cancer Research, Tokyo, Japan; d Center for Advanced Medical Development, Cancer Institute Hospital of Japanese Foundation for Cancer Research, Tokyo, Japan; e Division of Gynecologic Oncology, Cancer Institute Hospital of Japanese Foundation for Cancer Research, Tokyo, Japan; f Division of Hepato-Biliary-Pancreatic Medicine, Cancer Institute Hospital of Japanese Foundation for Cancer Research, Tokyo, Japan; g Division of Gastroenterological Chemotherapy, Cancer Institute Hospital of Japanese Foundation for Cancer Research, Tokyo, Japan; h Division of Clinical Genetic Oncology, Cancer Institute Hospital of Japanese Foundation for Cancer Research, Tokyo, Japan; i Division of Pathology, Cancer Institute, Japanese Foundation for Cancer Research, Tokyo, Japan; j Division of Pathology, Cancer Institute Hospital, Japanese Foundation for Cancer Research, Tokyo, Japan; k Pathology Project for Molecular Targets, Cancer Institute, Japanese Foundation for Cancer Research, Tokyo, Japan; l Project for Development of Innovative Research on Cancer Therapeutics, Cancer Precision Medicine Center, Japanese Foundation for Cancer Research, Tokyo, Japan; m Project for Immunogenomics, Cancer Precision Medicine Center, Japanese Foundation for Cancer Research, Tokyo, Japan

**Keywords:** Rare malignancy, Comprehensive genomic profiling, Druggable alterations, Genome-driven treatment

## Abstract

Without a current standard of care, patients with rare malignancy are subjected to precision oncology with next-generation sequencing to identify a course of treatment. We sought to establish the clinical relevance of comprehensive genomic profiling (CGP) among patients with rare malignancy. Rare malignancy was defined using the Rare Cancers in Europe definition (<6 cases per 100,000 individuals). We analyzed gene mutations, fusions, tumor mutational burden (TMB), and microsatellite instability (MSI) status. Level A gene alterations, categorized using Clinical Interpretations of Variants in Cancer and MD Anderson Knowledge Base for Precision Oncology, were considered druggable. Rare malignancy accounted for 149 (45%) cases, with female genital cancers (32%) most common. Among the rare malignancy cases, we identified a lower frequency of mutation in *TP53* (41% vs. 60%, *P*<0.001), *KRAS* (13% vs. 43%, *P*<0.001) and *APC* (3% vs. 25%, *P*<0.001), and a higher frequency of *ARID1A* mutation (14% vs. 6%, *P*=0.03), as compared with common malignancies. TMB-high and MSI-high cases were found in 8% and 2% of cases, respectively. Druggable alterations were detected in 37 patients with rare malignancy; this percentage tended to be higher than that for patients with common malignancies (25% vs. 17%, *P*=0.08). Common druggable alterations were *BRAF* V600E, *ERBB2* amplification, *PIK3CA* E542K, and *BRCA1/2* variant. Five of the 37 patients with druggable alterations received genome-driven treatment. There was no significant difference in overall survival between the rare and common malignancy groups. Our results provide clues for future clinical development and treatment success among Japanese patients with rare cancers.

## Introduction

Rare malignancy comprises a heterogenous group of about 200 types of cancers [1], and is largely divided into cancer within cancer-rare organs and rare cancer within cancer-common organs [1]. Although there are several criteria, the Rare Cancers in Europe (RARECARE) project, which defines rare malignancy as cancer with an annual incidence of <6 per 100,000 people, is pervasive worldwide [2,3]. In the USA, rare malignancy is defined as having an incidence of <15 per 100,000 people [4], and ‘ultrarare cancer’ has been proposed to describe malignancy with an incidence of <2 per 100,000 people [5]. Most (>70%) cancers categorized as rare have an extremely low annual incidence (<0.5 per 100,000 people), accounting for about 20% to 25% of all cancer diagnoses [6]. Regarding prognosis, an epidemiological study by the RARECARE project showed unfavorable overall survival (OS) rates among patients with rare malignancies (5-year OS rate: 47% for rare malignancy vs. 65% for common malignancy); this non-negligible frequency and poor prognosis highlight the need for clinical development for rare malignancies.

At present, several tumor agnostic treatments are approved: pembrolizumab is approved as an immune checkpoint inhibitor (ICI) for solid cancers with microsatellite instability (MSI)-high or mismatch repair deficiency and cancers with tumor mutational burden (TMB)-high; and larotrectinib or entrectinib are used as tropomyosin-related kinase (TRK) inhibitors for NTRK fusion-positive solid cancer [7], [8], [9]. These emerging therapies are attractive for treating rare malignancies where the development of conventional organ-based pharmaceutics by companies is generally inactive. In considering the clinical applications of ICI and TRK-inhibitors, comprehensive genomic profiling (CGP) is used as a companion diagnostic test.

Not surprisingly then, novel treatments for advanced rare malignancy have been left behind, and therapeutic options are limited when compared with the treatment options and continued development of drugs against common malignancies. As such, detecting druggable gene alterations using next-generation sequencing (NGS) offers a practical, relevant way to increase therapeutic options for patients with rare malignancy. When discussing the clinical utility of CGP for rare malignancy, existing data are scarce [10,11]. In this study, we focus on revealing druggable gene alterations among Japanese patients with rare malignancy to obtain referential information to inform future clinical trials.

## Methods

### Study overview and data collection

**Figure 1:**
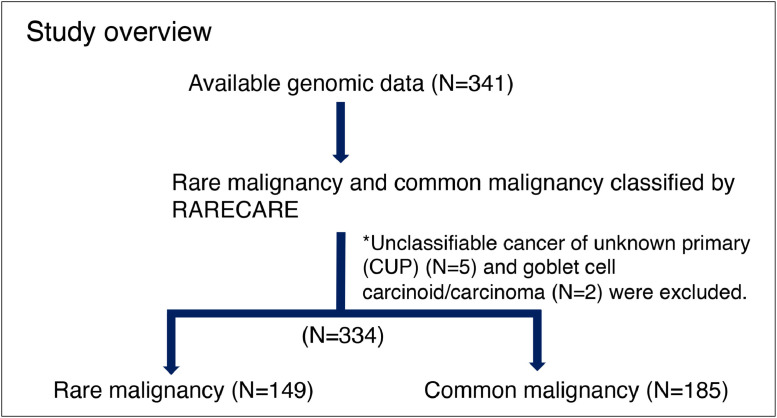
Study overview. A total of 341 patients with any malignancy who underwent CGP were reviewed. Based on the RARECARE definition (annual incidence of < 6 per 100,000), respective cases were categorized into either common malignancy or rare malignancy. Seven cases who were not listed in RARECARE data were excluded from the analysis. RARECARE, Rare Cancers in Europe.

This study was reviewed and approved by the Institutional Review Board of the Japanese Foundation for Cancer Research (JFCR) (NO. 2021-GA-1075) and conducted in accordance with the guidelines established by the Helsinki Declaration. The study design is outlined in Figure 1. We retrospectively reviewed 341 patients with any cancer who underwent CGP covered by Japanese insurance in the Department of Genomic Medicine, Cancer Institute Hospital, JFCR (Tokyo, Japan) between 2019 and 2021. Both male and female patients were included, and patient age ranged from 13 years to 84 years. Cases with material unsuitable for genomic analysis, and those with liquid biopsy samples only were excluded from this study. Based on the RARECARE definition, cases were categorized as either common malignancy, with annual incidence of ≥6 per 100,000, or rare malignancy, with the incidence of <6 per 100,000 [2]. Cancers for which the annual incidence was not listed in previous literature, such as unclassified cancers of unknown primary and goblet cell carcinoid/carcinoma, were excluded [2,3]. Genomic alterations and survival data were compared between common malignancy and rare malignancy groups. The reviewed data included primary organ, genomic alterations (gene variants, gene amplifications, gene fusions, TMB and MSI status), and overall survival (OS). OS was calculated as the interval from the time of CGP testing to death from any cause or the last follow-up.

### Genomic alteration detected by CGP and interpretation of druggable alteration

Two types of CGP (FoundationOne CDx [F1CDx] and OncoGuide NCC Oncopanel System [NCC Oncopanel]) were commercially available in Japan from 2019 as hybridization capture-based targeted NGS. Of the 341 patients, samples from 334 patients were subjected to F1CDx, with the remaining 7 samples subjected to NCC Oncopanel. F1CDx detects substitutions, insertion/deletion alterations, copy number alterations and gene rearrangements across 324 genes, as well as genomic signatures, such as MSI and TMB [12]. Genomic DNA was isolated from formalin-fixed, paraffin embedded (FFPE) tumor tissue specimens. Targeted sequencing was performed using a HiSeq 4000 system (Illumina, San Diego, CA, USA). In comparison, the NCC Oncopanel detects substitutions, insertion/deletion alterations, copy number alterations, and gene rearrangements in 124 genes [13]. Unlike for F1CDx, genomic DNA for the NCC Oncopanel was isolated from FFPE tumor tissue specimens and peripheral blood samples as a normal reference; this provides a way to differentiate somatic mutations from germline mutations. Sequencing was performed using the NextSeq 550Dx system (Illumina). For both F1CDx and the NCC Oncopanel, specimens needed to have tumor cell proportions of at least 20%. The gene lists for these tests are described in Supplementary Table 1. Further technical information about the CGP assay and bioinformatics analysis can be found elsewhere [14,15].

Pathogenicity for each identified variant was judged based on the patient's report from the Center for Cancer Genomics and Advanced Therapeutics (C-CAT). The potential druggability of each variant was accessed using the Clinical Interpretations of Variants in Cancer (CIViC) (https://civicdb.org/home) and MD Anderson Knowledge Base for Precision Oncology (https://pct.mdanderson.org/#/) databases. In this study, gene alterations were only interpreted as druggable if they were categorized as Level A by both databases. (Level A refers to gene alterations that fall into a clinical consensus in human medicine [CIViC] and have an FDA-approved agent along with a specific biomarker [MD Anderson Knowledge Base for Precision Oncology].) Gene alterations were categorized by referring to the malignancy type. Because our analysis targeted only rare malignancies, obtaining high-level evidence was deemed unrealistic due to the lack of robust global data. Therefore, we did not consider malignancy type in our classification and concentrated on the type of variant. TMB was divided into 3 categories (<10 mutations/megabase (mut/Mb), 10-20 mut/Mb, and ≥20 mut/Mb), and TMB-high was defined as ≥10 mut/Mb, on par with current FDA labels of pembrolizumab [7].

## Statistical analysis

Differences in categorical variables between groups were analyzed using Fisher's exact test. Survival curves were estimated using the Kaplan–Meier method, and *P-*values were calculated using the log-rank test. Effects were considered statistically significant at a two-sided *P* < 0.05. All statistical analyses were performed using EZR (v.1.4.1; Saitama Medical Center, Jichi Medical University, Shimotsuke, Japan), which is based on R and R commander [16]. Due to the retrospective nature, randomization or blinding was not performed in this study.

## Results

### Frequency of rare malignancy and primary organ distribution

**Figure 2:**
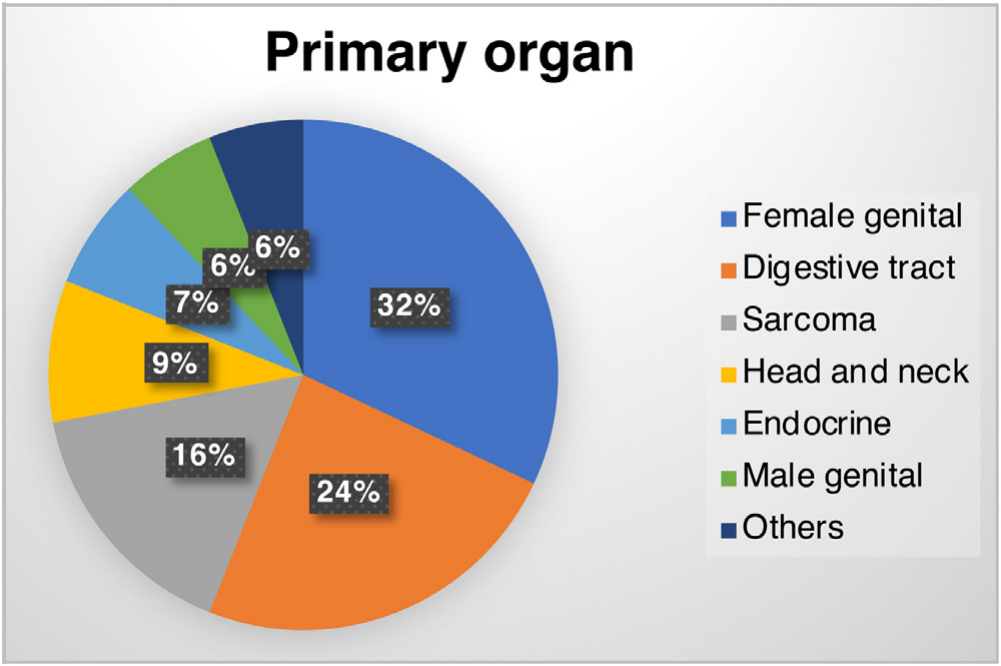
Primary organ distribution for 149 patients with rare malignancy.

Five cases with cancer of unknown primary (CUP) and 2 cases with goblet cell carcinoid/carcinoma were unclassifiable and excluded from further analysis. The remaining 334 cases were divided into either rare or common malignancy groups. Rare malignancy accounted for 149 (45%) of the 334 cases, with female genital cancers (32%) most common, followed by digestive cancers (24%), sarcoma (16%), and others (28%) (Figure 2). In terms of detailed malignancy type, ovarian cancer (19%), sarcoma (16%), and bile duct cancer (15%) were major cancers included within the rare malignancy group, versus colorectal cancer (28%), pancreatic cancer (26%), and breast cancer (18%) in the common malignancy group (Supplementary Table 2).

### Profile of gene mutations, gene amplifications and gene fusion in rare malignancy cases

**Figure 3:**
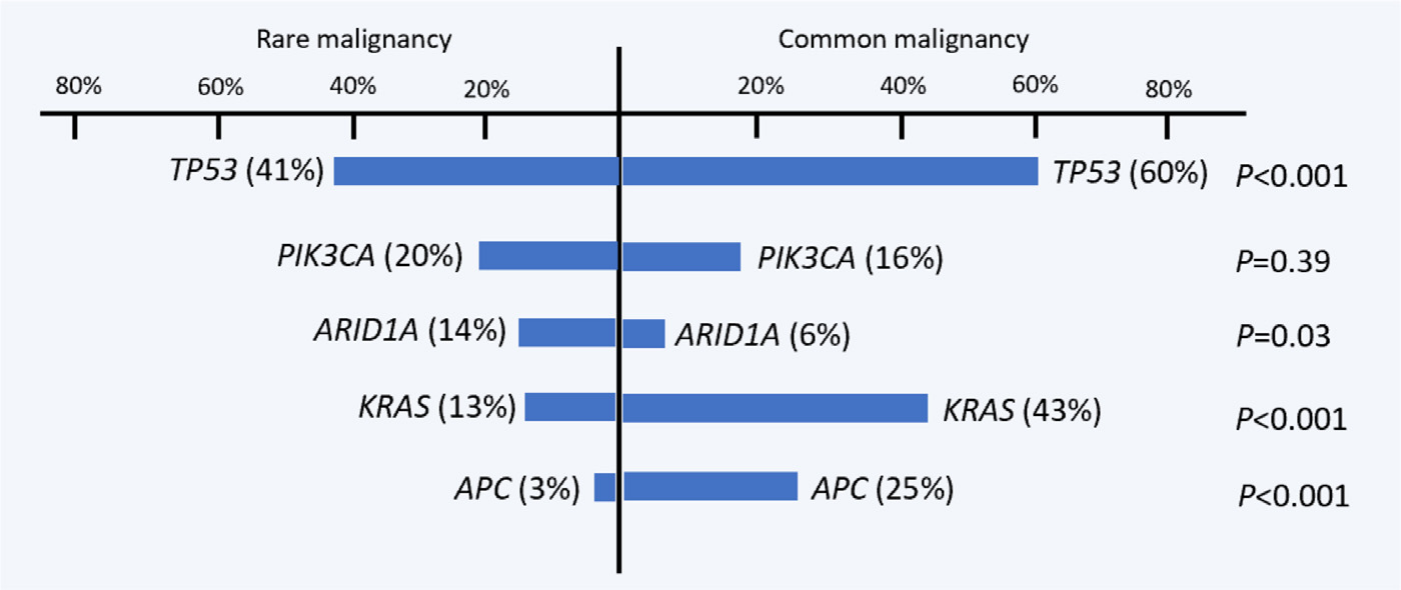
Gene mutation prevalence in rare malignancy and common malignancy subgroups.

Pathogenic/likely-pathogenic variants were identified in 123 cases of rare malignancy, which was significantly lower than that for the common malignancy cohort (83% vs. 95%, *P* <0.001). The predominantly mutated genes are shown in Figure 3. Compared with the common malignancy group, the rare malignancy group had a significantly lower frequency of *TP53* mutation (60% vs. 41%, *P*< 0.001), *KRAS* mutation (43% vs. 13%, *P*< 0.001) and *APC* mutation (25% vs. 3%, *P*< 0.001). On the other hand, the rare malignancy group had a higher prevalence of *ARID1A* mutation (14% vs. 6%, *P*= 0.03). *ERBB2* amplification was detected in 9 rare malignancy cases (median copy number, 8; range, 5-57). *EWSR1* gene fusion was found in 6 cases of sarcoma, and *FGFR2* fusion in 1 case of cholangiocarcinoma and 1 case of salivary duct carcinoma. Additionally, 1 spindle cell sarcoma case harbored *GOLGA5*-*RET* fusion. No germline mutations were detected among the 7 patients whose samples were subjected to the NCC Oncopanel (4 sarcoma, 1 melanoma, 1 prostate cancer and 1 pancreatic cancer cases).

### TMB and MSI status

Approximately 1.5% of the rare malignancy cases fulfilled the MSI-high status (1 case with adenocarcinoma from liver and 1 with urothelial carcinoma from kidney) (**Supplementary Table 3**). The median TMB was 3.0 (range, 0-76), and the proportion of cases with TMB ≥ 10 mut/Mb and ≥ 20 mut/Mb was 8% (*n* = 11) and 1% (*n* = 2) (1 case with ovary mixed adenocarcinoma and 1 with urothelial carcinoma), respectively. TMB in 2 MSI-high cases was over 10 mut/Mb (11 and 39 mut/Mb). The prevalence of MSI-high and TMB-high (≥ 10 mut/Mb) was comparable with that for common malignancy (*P* = 0.60 and 0.34, respectively).

### Druggable gene alterations and treatment

With reference to CIViC and MD Anderson Knowledge Base for Precision Oncology, druggable gene alterations were detected in 37 rare malignancy cases; this tended to be higher than that for common malignancy cases (25% vs. 17%, *P*= 0.08). *BRAF* V600E, *ERBB2* amplification, *PIK3CA* E542K, *BRCA1/2* variant, *KRAS* G12C, and *FGFR2* fusion were found in 7%, 6%, 5%, 4%, 3%, and 1.4% of cases, respectively (Table 1). *BRAF* V600E and *PIK3CA* E542K were predominantly associated with thyroid cancer and ovarian cancer, respectively, whereas *ERBB2* amplification, *BRCA1/2* variant, and *KRAS* G12C were widely detected across various cancer types. When we included MSI-high and TMB-high cases, the percentage increased from 25% to 31%. Among the 37 cases with druggable alterations, 5 patients received a genome-driven treatment (Table 2). Sotorasib was administered to 2 patients with *KRAS* G12C (chondrosarcoma and high-grade serous adenocarcinoma derived from fallopian tube), tucatinib plus trastuzumab to 1 patient with *ERBB2* amplification (ovarian clear cell adenocarcinoma), encorafenib plus binimetinib to 1 patient with *BRAF* V600E (thyroid papillary cancer), and FGFR2 inhibitor plus anti-PD-L1 antibody to 1 patient with *FGFR2*-*FOXP1* gene fusion (salivary duct carcinoma). None of the remaining 32 patients received a genome-driven treatment. This was chiefly because they were already using a proposed reagent as clinical practice (*n* = 6), were receiving present treatment without disease progression (*n* = 3), or they exhibited a deterioration in performance status in activities of daily living (*n* = 5) or organ dysfunction (*n* = 2).

**Table 1:** Druggable gene alterations detected in rare malignancy.

Gene alteration	Potential agent	Patient number	Type of malignancy
BRAF V600E	Vemurafenib, dabrafenib, encorafenib	10 (7%)	Thyroid (n=9), ovary (n=1)
ERBB2 amplification	Trasutuzumab, tucatinib, neratinib, lapatinib, trastuzumab emtansine, trastuzumab deruxtecan	9 (6%)	Ovary (n=4), bile duct (n=3), neuroendocrine (n=1), salivary gland (n=1)
PIK3CA E542K	Alpelisib	7 (5%)	Ovary (n=5), uninary tract (n=1), uterine cervix (n=1)
BRCA 1/2 variant	Olaparib	6 (4%)	Larynx (n=1), salivary gland (n=1), breast (n=1), ovary (n=1), urinary tract (n=1), bile duct (n=1)
KRAS G12C	Sotorasib	4 (3%)	Bile duct (n=1), ovary (n=1), sarcoma (n=1), methothelioma (n=1)
FGFR2 fusion	Erdafitinib	2 (1.4%)	Bile duct (n=1), salivary gland (n=1)
FGFR3 G370C	Erdafitinib	1 (0.7%)	Urinary tract (n=1)
FGFR3 R248C	Erdafitinib	1 (0.7%)	Urinary tract (n=1)

**Table 2:** Genome-driven treatment for patients with druggable alterations.

Patient ID	Age	Sex	Primary organ	Pathological diagnosis	Gene variant	Gene amplification	Gene fusion	Druggable alteration	Treatment Regime
1	63	F	Ovary	Clear adenocarcinoma	PPP2R1A P179L	ERBB2	None	ERBB2	Tucatinib plus trastuzumab
2	76	F	Fallopian tube	High-grade serous adenocarcinoma	KRAS G12C	None	None	KRAS G12C	Sotorasib
3	36	M	Sacrum	Chondrosarcoma	KRAS G12C	None	None	KRAS G12C	Sotorasib
4	50	F	Thyroid	Papillary carcinoma	BRAF V600E	None	None	BRAF V600E	Encorafenib plus binimetinib
5	64	M	Salivary gland	Carcinoma	PIK3CA H1047R	None	FGFR2-FOXP1	FGFR2-FOXP1	FGFR2 inhibitor plus anti-PD-L1 antibody

### Genomic characteristics in ultrarare malignancy

Of the 341 patients analyzed, 113 (33%) met the criteria of ultrarare malignancy with an annual incidence of <2 per 100,000 people. The common cancer types were sarcoma (*n* = 24), bile duct cancer (*n* = 23), ovarian cancer (including fallopian tube cancer and peritoneal cancer) (*n* = 19), and urothelial cancer (*n* = 9). While 12 ovarian cancer cases with adenocarcinoma were excluded from this category, all fallopian tube cancer cases were included, regardless of histology. The percentage of patients with druggable alterations based on the above two databases was 19%, and 24% of cases were classified as MSI-high or TMB-high.

### OS among in rare and common malignancies

**Figure 4:**
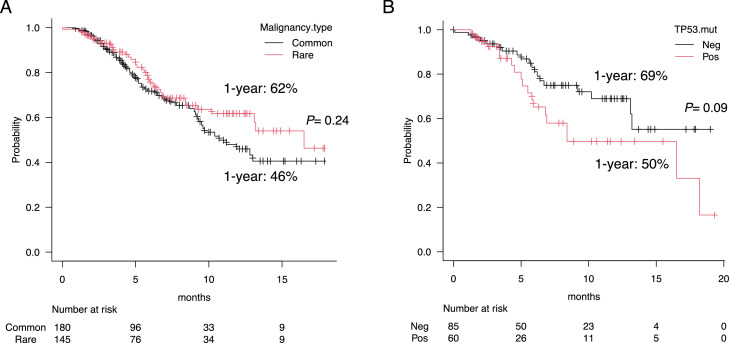
(A) Overall survival (OS) curves in rare malignancy (*n* = 145) and common malignancy (*n* = 180) subgroups. (B) OS curves in rare malignancy cases who harbored a *TP53* mutation (*n* = 60) and those without a mutation (*n* = 85).

The survival curves for the rare and common malignancy groups are shown in Figure 4A. The median follow-up duration for survivors was 5.7 months. The 1-year OS rate was 62% for those with rare malignancies and 46% for those with common malignancies; there was no significant difference for OS between the two groups (*P*= 0.24). The subgroup analysis for rare malignancy showed that OS was not significantly different between patients with ultrarare malignancy (*n* = 110) and those who were excluded from this category (*n* = 35) (62% vs. 66%, *P* = 0.81). As a next step, we analyzed the clinical impact of gene mutations on OS among the rare malignancy cases. Cases with *TP53* mutation tended to have worse OS than those without (1-year OS: 50% vs. 69%, *P* = 0.09) (Figure 4B), and there was no significant difference in OS between the mutated and unmutated cases for *KRAS, PIK3CA*, or *ARID1A* (1-year OS: 51% vs. 63% for *KRAS, P* = 0.18; 61% vs. 62%, *P* = 0.82 for *PIK3CA*; 47% vs. 63%, *P* = 0.59 for *ARID1A*).

## Discussion

The present work compared the genomic features and clinical outcomes between rare malignancy and common malignancy cohorts; such a direct comparison for CGP data is scarce worldwide. There are several important findings from this study. First, rare malignancy accounted for 45% of all cases, which is higher than that observed in previous epidemiologic studies (24%) [2,3]. This higher incidence might reflect the unmet needs of current practice for rare malignancy, wherein clinicians use CGP as a way to expand upon the available therapeutic options for patients.

Second, *TP53* mutation was the most commonly mutated gene (41%), followed by *PIK3CA* (20%), *ARID1A* (14%), and *KRAS* (13%) (Figure 3). Here, the prevalence of several of these mutated genes was different between the two cohorts. In the MD Anderson Cancer Center (MDACC) study, mutations were conspicuously detected in *TP53* (23%), *KRAS* (10%), and *PIK3CA* (9%) [10], whereas, in the University of California San Diego (UCSD) study, gene alteration prevalence was 46% for *TP53*, 12% for *CDKN2A/B*, 12% for *RB1*, and 12% for *KRAS* [11]. While both our study and the aforementioned two studies consistently highlight *TP53* as the major mutated gene, the high proportion of *ARID1A* mutation in our study is in contrast with the findings of the other two studies. This discrepancy may be explained by differences in the distribution of cancer type within the rare malignancy cohort particular to certain populations. Indeed, ovarian cancer, the most dominant in our rare malignancy population (19%), accounted for 52% of all *ARID1A*-mutated cases; however, is found at lower frequency in the other populations. The percentage of MSI-high and TMB-high (≥20 mut/Mb) in our rare malignancy cohort was 2% and 1%, respectively, which are lower than 3% of TMB ≥20 mut/Mb in the MDACC study [10].

Third, we found that 25% of patients with a rare malignancy harbored a potentially druggable alteration. When this was confined to ultrarare malignancies, the rate was still about 20%. In the MDACC study, the rate of any potentially actionable alteration was 38%, with PI3K/AKT, MAP kinase and NOTCH pathway alterations, *KRAS* mutations, *FGFR* and *NTRK* alterations, *SOX2* amplification, and mutations in germline-associated genes (*VHL, TSC2, NF1, NF2*) identified as druggable targets [10]. Based on the NGS data, 13 patients had been administered targeted therapy: 5 patients with the *BRAF* V600E variant had received a BRAF inhibitor treatment, and 7 patients with PI3K/AKT pathway aberrations had received an either mTOR inhibitor, AKT inhibitor, or PI3K inhibitor treatment. Contrastingly, in the UCSD study, 93% of the patients harbored a theoretically actionable alteration based on genomic or protein markers [11]; this is a considerable gap in the druggability rate when compared with our study (25%). This may be caused by differences in the definition of druggability or the inclusion of different types of cancers. We used an unequivocal and rigid definition for inclusion (level A based on CIViC and MD Anderson Knowledge Base for Precision Oncology), and thus our results are firm and practical in considering druggability. Requiring further consideration is that TMB and MSI status were not mentioned in the above two databases and were therefore excluded from the druggability rate calculation in our analysis. Including MSI-high or TMB-high cases raised the total druggability rate to 31%, which is comparable with the rate reported in the MDACC study (38%) [10].

As listed in Table 1, the agents for druggable alterations are BRAF inhibitor for the patients with *BRAF* V600E variant, anti-HER2 antibody for those with *ERBB2* amplification, PI3Kα inhibitor alpelisib for those with the *PIK3CA* E542K variant [17], PARP inhibitor for the *BRCA1/2* variant, *KRAS* G12C inhibitor sotorasib for the *KRAS* G12C variant [18], and pan-FGFR inhibitor erdafitinib for the *FGFR2* gene fusion or specific *FGFR3* variants [19]. The *BRCA1/2* variant and the *KRAS* G12C mutation were detected regardless of malignancy type. Salem et al. analyzed approximately 79,000 samples with various types of cancers and showed that 17% of cases had *KRAS* mutations, including the *KRAS* G12C mutation (2%) [20]. *KRAS* G12C was widely detected across the primary organ (9% for non–small-cell lung cancer, 3.9% for appendiceal cancer, 3.2% for colorectal cancer, 1.6% for tumor of unknown origin, 1.4% for small bowel cancer, and 1.3% for pancreatic cancer) [20]. In a phase 2 trial for patients with lung cancer harboring the *KRAS* G12C mutation, sotorasib treatment resulted in an overall response rate (ORR) of 37%. In terms of *FGFR* aberrations, a large-scale study of 4,853 solid tumors showed that 7% of cases had some *FGFR* aberration, with *FGFR2* and *FGFR3* aberrations accounting for 19% and 26% of all *FGFR* aberrations, respectively [21]. *FGFR* aberrations were highest among urothelial carcinoma (32%), followed by breast carcinoma (18%) and endometrial adenocarcinoma (12%). A phase 2 trial of erdafitinib for patients with locally advanced or metastatic urothelial carcinoma who harbored *FGFR* alterations showed favorable ORR (40%). Additionally, in our cohort, selpercatinib was administered to a patient with spindle cell sarcoma harboring a *GOLGA5*-*RET* gene fusion. Although this gene fusion was categorized as level A with MD Anderson Knowledge Base for Precision Oncology, it was unclassifiable in CIViC, and this patient was comprehensively excluded from among the druggable cases. *GOLGA5* is a RET proto-oncogene interacting gene; its gene fusion pattern is characteristic of patients with papillary thyroid carcinomas who were exposed at young age to radioiodine released from the Chernobyl reactor [22,23].

In our analysis, only 14% of patients with any druggable alterations could receive genome-driven treatment. This percentage could be improved with appropriate timing of CGP testing, particularly because performance status deterioration among these subjects complicates the use of suggested agents. Tight cooperation with a phase 1 trial team is also indispensable. Furthermore, CGP in Japan is, in principle, covered by insurance, and suitable candidates for CGP are strictly determined in the package insert. The situation might be different in the USA, where more patients are able to access to CGP. The German Cancer Consortium has recently reported successes with genome-driven treatments [24]. In their approach, therapeutic decisions were guided using whole-genome/exome and RNA sequencing data, with the overall response rate improving from 16% (last systemic therapy before genomic/transcriptomic analysis) to 24% (molecularly informed treatment). The authors considered that the promising outcome was partly explained by integrating RNA sequencing data with DNA sequencing data. For these cases, the progression-free survival (PFS) ratios (PFS in molecularly informed treatment/PSF in standard therapy) were >1.3 in 36% and >1.5 in 31% of patients.

Another focus is to determine whether there is any difference in prognosis between patients with rare malignancy and those with common malignancy. Unlike previous data, our rare malignancy group had no adverse impacts for OS. It should be noted that our investigation targeted only select patients who underwent genomic analysis in our institution. Additionally, OS was calculated from the time of CGP testing but not the initial diagnosis. Another possible explanation is the relatively high proportion (26%) of intractable pancreas cancer in our common malignancy cohort.

There were several limitations in this study. The patients were from a single institution, and therefore the sample size was limited. In addition, although we reviewed all cases since the establishment of our Genomic Medicine Department, the follow-up duration for OS was short (<6 months). Third, detailed information regarding the clinical efficacy of genome-driven treatment is unavailable and, in many cases, associated with ongoing trials. Finally, it should be noted that incidence rates for several malignancies are different between Western countries and Asian countries (Japan included). For example, esophageal squamous cell carcinoma or hepatocellular carcinoma, which is classified as rare malignancy based on the RARECARE definition is common in Japan (14. 2 for esophageal carcinoma and 19.2 for hepatocellular carcinoma per 100,000 people, respectively) [25].

## Conclusions

We show that rare malignancies among patients in Japan have specific genomic features, with around 25% of the rare malignancy cases in our cohort harboring druggable alterations (*BRAF* V600E, *ERBB2* amplification, *PIK3CA* E542K, and *BRCA1/2* variant) for which approved agents exist. These specific genomic features will help to provide clues for the future clinical development and assessment of treatments for patient care, and assist the identification of ways to deliver precise medical care to these patients in alignment with CGP.

## Data availability statement

The data generated in this study are available within the article and its supplementary data files.

## Author's contributions

A.O., N.H., K.K., and S.T. conceived and designed the study, and wrote the paper; I.F., M.Y., M.Y., A.K., E.S., A.U., A.T., K.T., and S.M. reviewed, and revised the paper; A.O., and N.H. acquired, analyzed, and interpreted data, and performed statistical analysis; All authors read and approved the final manuscript.

## Appendix Supplementary materials

Image, application 1

Image, application 2

Image, application 3
